# First-Principles Atomistic Thermodynamics and Configurational Entropy

**DOI:** 10.3389/fchem.2020.00757

**Published:** 2020-12-03

**Authors:** Christopher Sutton, Sergey V. Levchenko

**Affiliations:** ^1^Department of Chemistry and Biochemistry, University of South Carolina, Columbia, SC, United States; ^2^Skolkovo Innovation Center, Skolkovo Institute of Science and Technology, Moscow, Russia

**Keywords:** statistical mechanics, cluster expansion, chemical potential, phase diagram, atomistic thermodynamics method, configurational entropy

## Abstract

In most applications, functional materials operate at finite temperatures and are in contact with a reservoir of atoms or molecules (gas, liquid, or solid). In order to understand the properties of materials at realistic conditions, statistical effects associated with configurational sampling and particle exchange at finite temperatures must consequently be taken into account. In this contribution, we discuss the main concepts behind equilibrium statistical mechanics. We demonstrate how these concepts can be used to predict the behavior of materials at realistic temperatures and pressures within the framework of atomistic thermodynamics. We also introduce and discuss methods for calculating phase diagrams of bulk materials and surfaces as well as point defect concentrations. In particular, we describe approaches for calculating the configurational density of states, which requires the evaluation of the energies of a large number of configurations. The cluster expansion method is therefore also discussed as a numerically efficient approach for evaluating these energies.

## 1. Introduction

At finite temperatures (*T* > 0 K), where functional materials typically operate, atoms move randomly in all directions due to the energy provided by heat sources. Moreover, a material is almost always in contact with a gas (or liquid), and can exchange particles with its environment. The system (“material plus environment”) therefore constantly samples its configurational space with a finite probability to eventually overcome barriers that separate the minima on the potential-energy surface (PES). If the barriers are not very high and/or the time for the system to explore its configurational space is sufficiently large, the system will end up in a state of thermodynamic equilibrium. In the hypothetical situation when the system is efficiently cooled down to *T* = 0 K, it will eventually relax from an arbitrary state to a local or global minimum on the PES, minimizing the internal energy at these conditions. A more realistic scenario, however, is a case where the material interacts with a heat bath (a practically infinite energy reservoir, e.g., Earth's atmosphere), which keeps the temperature of the system constant. In this case, for purely statistical reasons, the system will tend to spend most of its time (i.e., will have a high probability to be found) in the parts of the PES with many local minima of a similar energy (i.e., with a high density of states), provided the energies of these states are not much higher than the global minimum. This effect can be formalized by introducing the concepts of entropy and free energy: at a finite temperature, the system tends to minimize its free energy rather than its internal energy because entropy is maximized.

**Figure 1:**
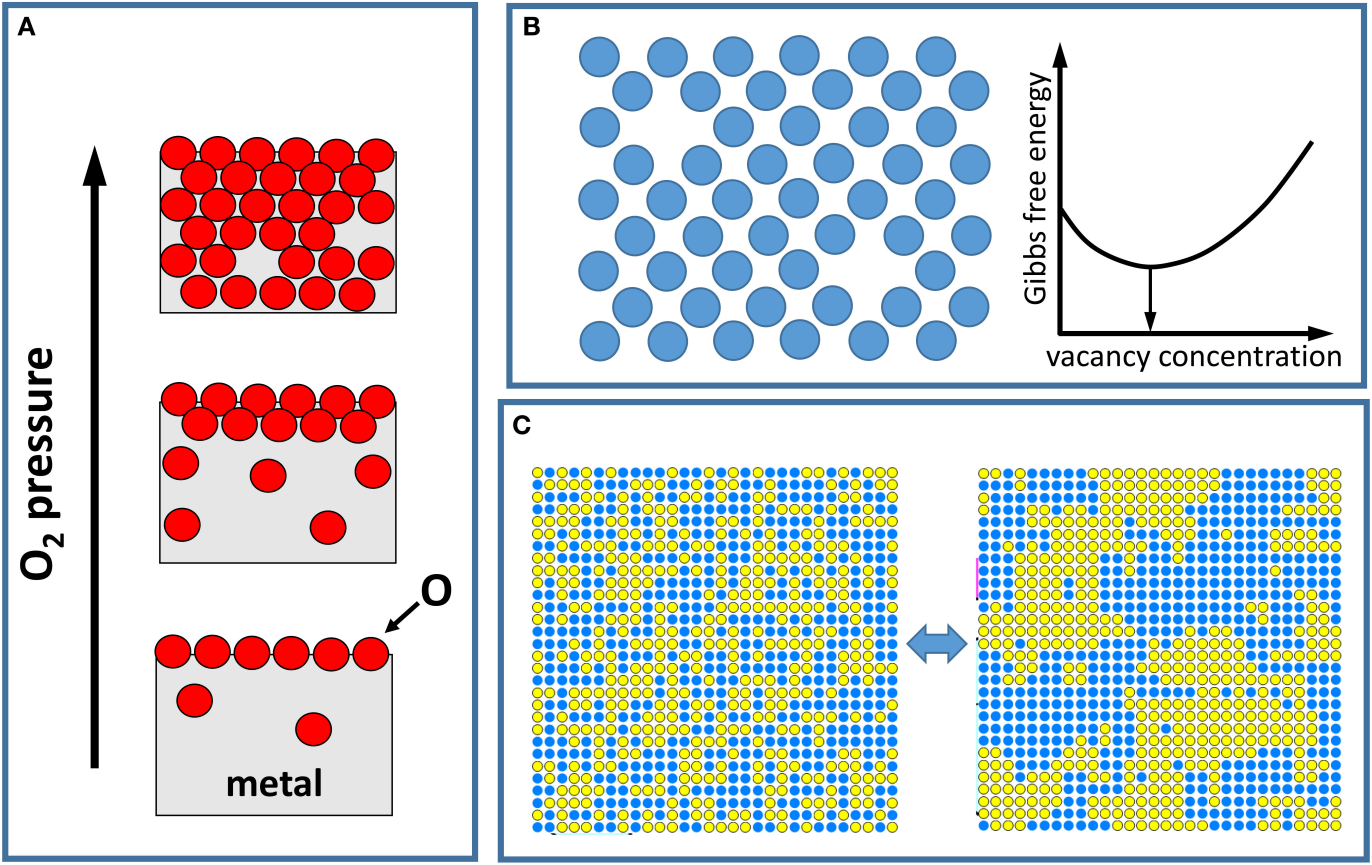
Pictorial demonstration of phenomena driven by thermodynamics. **(A)** Illustration of the oxidation of a metal surface as the oxygen pressure is increased, where first a change in the oxygen content at the surface and then subsequently in the bulk metal is observed. A (very) small amount of oxygen can be present in the bulk even at low pressures, while some O atoms may be missing once the oxide is formed at higher pressures. **(B)** Formation of defects. A generic atomic/molecular lattice model (e.g., of a crystal surface) with missing atoms/molecules illustrates a random distribution of vacancy defects. The white curve shows the dependence of the Gibbs free energy of the system with the defect concentration. In thermodynamic equilibrium, the concentration of defects minimizes the free energy. **(C)** Illustration of an order disorder phase transition for a binary alloy. At higher temperatures, the alloy components are randomly mixed (left image) due to thermal population of quasi-degenerate configurational states. As the temperature is decreased, the attractive interaction between atoms within each alloy component causes separation of the components (right image).

The entropy can be viewed as a measure of the (quasi)degeneracy of the states of a system that are accessible at a given temperature. The distinguishable states of a material contributing to entropy can vary in origin, as they correspond to different degrees of freedom. For example, vibrational and electronic states give rise to the corresponding entropic contributions in solid-state materials, whereas rotational and translational contributions are important for a gas. An additional important component in determining the concentration of point defects and order-disorder phase transitions is the configurational entropy, which is associated with the degeneracy of different atomic/molecular configurational states (see Figure 1).

In solid-state alloys, the configurational disorder corresponds to all the distinct ways the lattice sites can be occupied at a specific concentration. As an example, consider a solid-state compound *A* with a small amount of impurities *B*. If species *A* and *B* tend to form a stable compound *AB* (or any other stoichiometry), the ground state of this system at *T* = 0 K will be the (ordered) compound *AB* embedded into *A*. At finite temperatures, however, the component *B* prefers to be randomly distributed in *A* because there are many more configurations with *B* distributed than for the single chunk of *AB*, resulting in a higher entropy. Depending on several factors, such as the concentration of *B*, the energy gain due to the formation of *AB*, and the temperature, the entropy term may therefore drive the system to where *B* is randomly dispersed in the material despite the stability of *AB* at *T* = 0 K. Similar considerations are applicable to adsorbates on surfaces (see Stampfl et al., 1999; Reuter et al., 2005; Capdevila-Cortada and López, 2016; Goldsmith et al., 2018 and references therein). A slightly different situation occurs when the concentration of *B* can vary due to the presence of a reservoir of *B*. This is a typical situation for defects (e.g., oxygen vacancies in a material exposed to air). In the simplest case when the interaction between defects *B* can be neglected and the formation of each defect consumes energy the equilibrium concentration is determined entirely by the entropy.

To calculate the effects of configurational entropy on stability, the configurational density of states (DOS) has to be evaluated. If there is no way to determine *a priori* which configurations fall within a given energy range, an extensive sampling of the configurational states is required. At the same time, the relative energies of different configurations should be evaluated with an accuracy that is on the order of thermal energy *k*_B_*T* for a reliable prediction of thermodynamic properties. This poses a challenge for modern *ab initio* electronic-structure methods, such as density-functional theory (DFT). Although certain DFT approximations work well within their domain of applicability, the computational cost remains too high for sampling of the potentially very large configurational space. Numerically efficient methods that still maintain a high accuracy are thus needed.

Force fields (trained on either empirical or *ab initio* data) have been traditionally used to study thermodynamics of solid-state materials, molecules (e.g., proteins) and liquids (e.g., solutions). Parameterized force fields, however, typically retain an acceptable accuracy only within the narrow range of structures and compositions for which they were tuned. Recently, more flexible approaches have emerged based on machine learning (ML) that allows for the sampling of large materials spaces (Bartók et al., 2010; Thompson et al., 2015; Behler, 2016; Shapeev, 2017; Smith et al., 2017). Typically, however, ML models require a large number of training samples (i.e., structures where the energy is already known) before a sufficient accuracy can be achieved. In addition, issues with transferability and generalizability between significantly different structures could also be a problem with these methods (Sutton et al., 2019). Although addressing these issues is an active area of ongoing research (Sutton et al., 2020), we instead focus the discussion here on the cluster expansion (CE) method (Sanchez et al., 1984; de Fontaine, 1994; Ducastelle, 1994). The CE method has been widely used in calculating the thermodynamic properties of alloys because it is a numerically efficient approach for estimating the energy for practically all of the numerous configurational states of a specific lattice, given only a handful of initial DFT calculations (typically <100) for training the model.

In this contribution, we begin with an introduction to the basic concepts of thermodynamics in section 2. This is followed in sections 3 and 4 by a brief description of computational methodologies for evaluating configurational density of states and other thermodynamic quantities, including several sampling techniques and the CE approach. In section 5.1, we compare the computed thermodynamic quantities from two different sampling methods for an exemplary alloy (CuAu). We then provide a discussion of the role of configurational disorder in point defect formation in section 5.2. Finally, construction of the surface phase diagram using first first-principles calculations is discussed in section 5.3.

## 2. Basic Concepts

Here, we only outline the basic concepts of statistical mechanics and thermodynamics to pave the way for understanding the methods and practical examples discussed in this contribution. A more detailed and general formulation can be found elsewhere, e.g., Hołyst and Poniewierski (2012). To begin let us consider a system of a large set of (interacting) particles (atoms and/or molecules) in contact with a thermostat (i.e., a large auxiliary system that can serve as a source or a sink of the heat, keeping the temperature of the system constant). The system can be a solid, liquid, gas, or any combination of these. Due to the statistical effects described in the introduction, the system will tend to minimize its free energy. If both the temperature *T* and volume *V* are constant, the system will minimize the Helmholtz free energy *F*:

(1) $$\begin{array}{l} F=U-TS. \end{array}$$

Instead of (or in addition to) a constant volume, if the pressure *p* is kept constant, the system tends to a state that minimizes the Gibbs free energy *G*:

(2) $$\begin{array}{l} G=U+pV-TS. \end{array}$$

These quantities are called thermodynamic potentials. In both cases, the number of atoms/molecules of each type is assumed to be constant as well. These particles are enclosed in a volume that is either kept constant or evolves so that the pressure is constant. If the system consists, for example, of a solid and a gas, the number of particles of each type in the solid can change, which must be accompanied by a corresponding change in the number of particles of the same type in the gas so that the total number of particles in the system (i.e., solid + gas) is constant.

According to statistical mechanics, all thermodynamic quantities, including the Gibbs free energy, internal energy, and entropy, can be expressed via a partition function *Z*:

(3) $$\begin{array}{l} Z=\int_{-\infty }^{\infty }\sigma \left(E\right){\text{exp}}^{-E/k_{\text{B}}T}dE, \end{array}$$

where σ(*E*) is the DOS (number of states per energy unit). In this form, the equation is applicable to both manifolds of continuous and discreet states, with the latter formally represented by Dirac δ functions in the DOS located at the energies of the discreet states. Knowing *Z* allows for various thermodynamic quantities to be calculated as follows:

(4) $$\begin{array}{l} U=k_{\text{B}}T^{2}\frac{\partial \text{ ln }Z}{\partial T},S=k_{\text{B}}\text{ ln }Z+k_{\text{B}}T\frac{\partial \text{ ln }Z}{\partial T},G=-k_{\text{B}}T\text{ ln }Z+pV. \end{array}$$

The DOS thus fully determines the thermodynamic properties of a system.

An important quantity is chemical potential that characterizes the system and needed for practical applications of Equation (2). Here, we focus on the constant (*T*, *p*) conditions since these are the most common constraints in well-controlled experiments and industrial applications. By *p* we imply a set of partial pressures *p*_*i*_ for each particle type *i* (in all examples in this contribution *i* enumerates different atomic species), each of which is kept constant. At these conditions, chemical potential of species *i* is defined as the derivative of *G* with respect to the number of particles of type *i*:

(5) $$\begin{array}{l} \mu_{i}={\left(\frac{\partial G}{\partial N_{i}}\right)}_{T,p,N_{j\neq i}}. \end{array}$$

In thermodynamic equilibrium, the chemical potential of each particle type has the same value everywhere in the system (i.e., either in the solid or in the gas).

From the extensivity of the free energy (i.e., the fact that it should change proportionally to the number of particles in the system) follows the Gibbs-Duhem relation:

(6) $$\begin{array}{l} G=\underset{i}{\sum }\mu_{i}N_{i}. \end{array}$$

This relation can be used to determine the stoichiometry of a part of the whole system in thermodynamic equilibrium. This describes realistic situations, such as a solid (bulk and/or surface) in equilibrium with a surrounding atmosphere. The free energy of the whole system can be written as the following:

(7) $$\begin{array}{l} G=G^{\left(1\right)}+\underset{i}{\sum }\mu_{i}N_{i}^{\left(2\right)}, \end{array}$$

where *G*^(1)^ is the free energy of phase 1 (e.g., a bulk solid or a surface), and $N_{i}^{\left(2\right)}$ is the number of particles of type *i* in the second phase (e.g., a gas). Here, we used Equation (6) for the second phase and the fact that chemical potentials in equilibrium are the same for both phases. Thus,

(8) $$\begin{array}{l} G=G^{\left(1\right)}+\underset{i}{\sum }\mu_{i}\left(N_{i}-N_{i}^{\left(1\right)}\right), \end{array}$$

where $N_{i}^{\left(1\right)}$ is the number of particles in phase 1. At this stage we assume that phase 2 is a reservoir large enough to set the chemical potential for the whole system, so that μ_*i*_ = const. Because *N*_*i*_ = const, the minimum of the total free energy *G* with respect to $N_{i}^{\left(1\right)}$ corresponds to the minimum of

(9) $$\begin{array}{l} \tilde{G}=G^{\left(1\right)}-\underset{i}{\sum }\mu_{i}N_{i}^{\left(1\right)}. \end{array}$$

The quantity $\tilde{G}$ is in fact a thermodynamic potential, corresponding to conditions of constant *T*, *p*, and μ (not *N*). It describes an open system that can exchange particles (i.e., atoms, molecules, or electrons) with a reservoir. The electronic chemical potential is often referred to as Fermi energy, although sometimes a distinction is made between these two concepts by specifying that the Fermi energy is the chemical potential at *T* = 0 K (Kittle, 2004).

The use of Equation (9) also requires knowledge of the chemical potentials of all particle types in the system. The experiments where (*T, p*) conditions for multiple species are carefully controlled are very rare (e.g., FactSage) (Bale et al., 2016). Assumptions on the values of chemical potentials of at least some of the species therefore have to be made for comparison with experimental results. This is done based on physical considerations, assuming reasonable reservoirs for the species. For example, for a binary metal oxide Me_*x*_O_*y*_ in an O_2_ atmosphere at constant *T* and *p*_O_2__, the chemical potential of the metal (Me) atoms is determined by the bulk oxide as the reservoir according to the Gibbs-Duhem relation:

(10) $$\begin{array}{l} g_{{\text{Me}}_{x}{\text{O}}_{y}}=x\mu_{\text{Me}}+y\mu_{\text{O}}, \end{array}$$

where *g*_Me_*x*_O_*y*__ is the Gibbs free energy of the bulk oxide per formula unit. For a ternary or a more complex oxide, this approach would only determine the chemical potential of the combination of metal species, but not each of them separately. Moreover, in an experiment, the species may be present in another form, e.g., as a component of another compound, which can then serve as a reservoir for that species. A common approach to addressing such issues is to study the whole range of chemical potentials and compare the predicted and experimentally detected changes in the system (phase transitions in particular) upon varying (*T, p*) conditions.

In some cases, it is reasonable to assume that the number of particles of a certain type is constant or changes negligibly for a range of conditions. For example, in metal or oxide alloys, when the minority metal species are not volatile at the conditions of interest. In this case, the free-energy differences between different configurations/compositions do not depend on the chemical potential of the particles of that type.

Finally, chemical potentials can be calculated self-consistently by minimizing the free energy under additional physical constraints, such as charge neutrality. This is a widely adopted approach for calculating concentrations of charged defects in solids, where the chemical potential of electrons is determined by the concentration and vice versa (Van de Walle and Neugebauer, 2004).

In the case of a gas-phase reservoir, the chemical potentials for the species whose temperature and pressure are controlled in an experiment can be calculated explicitly. This is particularly straightforward for an ideal gas, which is an accurate approximation for most gases at realistic temperatures and pressures. Furthermore, because the translational, electronic, rotational, vibrational, and nuclear (due to nuclear spin) degrees of freedom for each molecule in the gas can usually be decoupled, the partition function of the system is simply a product of partition functions for each molecule and each degree of freedom.^1^

After calculating of the partition function for each molecule, the chemical potential is obtained using Equation (5), with the expression of *G* in terms of the total partition function *Z*, and the ideal gas law *pV* = *Nk*_B_*T*:

(11) $$\begin{array}{l} \mu \left(T,p\right)=\frac{\partial }{\partial N}\left(-k_{\text{B}}T\text{ ln }Z+Nk_{\text{B}}T\right). \end{array}$$

Alternatively, the chemical potentials of common gases can be calculated from experimental data, for example the NIST-JANAF thermochemical tables (see Chase, 1998 and https://janaf.nist.gov/). Usually, data are reported only for a reference pressure *p*°. For an arbitrary pressure, the chemical potential can be calculated assuming the ideal gas behavior:

(12) $$\begin{array}{l} \mu \left(T,p\right)=\mu \left(T,p^{◦}\right)+k_{\text{B}}T\text{ ln }\left(p/p^{◦}\right). \end{array}$$

Another common practice is to introduce references for the chemical potentials. Implicitly, a reference is used whenever a numerical value for chemical potential is given. By introducing references $\mu_{i}^{\text{ref}}$, Equation (9) can be re-written:

(13) $$\begin{array}{l} \tilde{G}=\left[G^{\left(1\right)}-\underset{i}{\sum }\mu_{i}^{\text{ref}}N_{i}^{\left(1\right)}\right]-\underset{i}{\sum }\Delta \mu_{i}N_{i}^{\left(1\right)}, \end{array}$$

where $\Delta \mu_{i}=\mu_{i}-\mu_{i}^{\text{ref}}$. Note that this expression is equivalent to Equation (9) because we simply added and subtracted the same term. No observable, including the equilibrium values for $N_{i}^{\left(1\right)}$, can thus be affected by changing chemical-potential references. They are introduced for convenience, namely, to put the values of chemical potentials on a scale physically relevant for a given problem. For example, for a surface under an O_2_ atmosphere, a convenient reference for the oxygen chemical potential is μ_O_ = 1/2*E*_O_2__, where *E*_O_2__ is the total energy of an isolated oxygen molecule. Then the values of Δμ_O_ are of the order of an eV and reflect changes in the free energy (including the zero-point vibrational energy) per O atom of an O_2_ gas with temperature and pressure relative to the isolated O_2_ molecule. The zero-point energy can be also included in the reference. Interestingly, Δμ_O_ is less sensitive to the approximations in the electronic-structure method than μ_O_ (including 1/2*E*_O_2__) due to a cancellation of errors. Similarly, the value of the difference in the square brackets in Equation (13) acquires the meaning of a formation energy or enthalpy upon a proper choice of $\mu_{i}^{\text{ref}}$ and can be directly compared to an experimental value. In general, chemical-potential references can be temperature and/or pressure dependent.

The free energy *G*^(1)^ in Equation (9) can be calculated using an electronic-structure method, such as DFT. The resulting approach is called *ab initio* atomistic thermodynamics (Weinert and Scheffler, 1986; Kaxiras et al., 1987; Qian et al., 1988; Moll et al., 1998; Reuter and Scheffler, 2001, 2003a). In principle, this requires calculating the electronic, vibrational, and configurational DOS as well as the *pV* term (e.g., accounting for the change in volume of a solid due to formation of defects or adsorption of molecules at the surface). The *pV* term at realistic pressures can, however, usually be neglected (Reuter and Scheffler, 2001). In any case, such approximations must be carefully tested and used with caution (Valtiner et al., 2009). This can be done by calculating the contributions for representative systems or by using approximate models (Reuter and Scheffler, 2001).

The vibrational contribution to the free energy can be estimated by *ab initio* methods (Stoffel et al., 2010), requiring the calculation of the interatomic force constants in a large supercell, which is computationally demanding. An important approximation that significantly simplifies the calculation of vibrational contributions to the free energy in solids is the harmonic approximation. In this approximation, the vibrational free energy takes the form (Ghatak, 2005):

(14) $$\begin{array}{l} F_{\text{vib}}=\int_{0}^{\infty }\sigma_{\text{phonon}}\left(\omega \right)\left[\frac{\hbar \omega }{2}+k_{\text{B}}T\text{ ln }\left(1-e^{-\hbar \omega /k_{\text{B}}T}\right)\right]d\omega , \end{array}$$

where σ_phonon_(ω) is the phonon DOS. The σ_phonon_(ω) is available for several hundred compounds in the Phonon database (PhononDB) (Togo, 2015). The harmonic approximation usually works well at low to moderately high temperatures (i.e., below 1,500 K), unless the PES is strongly anharmonic (e.g., when weak bonds are present in the system). The contribution of anharmonicity to the free energy can be evaluated using effective harmonic Hamiltonians or, more accurately, molecular dynamics simulations (see Grabowski et al., 2019 and references therein). The latter can be achieved either via thermodynamic integration or via enhanced sampling (Abrams and Bussi, 2014; Ikebe et al., 2016; Zhou et al., 2019).

Although computationally demanding to account for, the vibrational contribution to the free energy has been shown to be important for describing the stability of solid-state alloys (Ozoli and Asta, 2001; van de Walle and Ceder, 2002; Fultz, 2010; Benisek and Dachs, 2015). A main contributor to the vibrational entropy is the variation in the bonding environment of the different lattice sites in a given material (Fultz, 2010). For a reaction that involves two different phases of an element (e.g., oxygen in a bulk oxide as the product formed from a gaseous oxygen reactant), the vibrational free energy of the reactants and products can differ significantly because of the change in the bonding between the atoms in these different states. This incomplete cancellation of the vibrational contributions to the free energy can grow with an increasing temperature and significantly impact the calculated stability of a material (Bartel et al., 2018). A cancellation of errors is most likely to occur when taking the *differences* in Gibbs free energies of compounds with similar interatomic bonding environments in the reactant and products; however, this should be carefully tested.

## 3. Configurational Entropy

In this section, we introduce approaches for estimating ensemble averages of materials parameters. In principle, ensemble averages can be obtained directly using molecular-dynamics simulations (see, e.g., Baron et al., 2006; Rick, 2006 and references therein). This is, however, computationally expensive and currently even unfeasible for a majority of practically relevant problems in materials science. Instead, here we discuss a few commonly used approaches, such as the Monte-Carlo Metropolis algorithm and the Wang-Landau algorithm, as well as the Bayesian method nested sampling, which has emerged recently as an approach to explore phase space in an unbiased way. In section 5.1, we provide a comparison of the Metropolis algorithm and nested sampling in modeling the order-to-disorder transition in the binary alloy, CuAu.

We note that the three methods discussed here are just to highlight a few different common approaches. Several other algorithms can be used to calculate thermodynamic properties, such as umbrella sampling (Berg and Neuhaus, 1992) and replica-exchange molecular dynamics (Swendsen and Wang, 1986). Moreover, an interesting approach that has been developed recently combines replica-exchange molecular dynamics with Monte Carlo steps, adding or removing atoms (Zhou et al., 2019). This allows for both the exploration of configurational and compositional spaces in a single framework and the incorporation of anharmonic contributions to the free energy, although at a high computational cost. Additionally, several fast stochastic optimization techniques have been developed to efficiently search for ground-state structures. Examples include simulated annealing (Kirkpatrick et al., 1983), genetic algorithms (Abraham and Probert, 2006; Oganov and Glass, 2006; Falls et al., 2016), basin hopping (Wales and Doye, 1997; Doye and Wales, 1998), and minima hopping (Goedecker, 2004). These approaches are not constrained to sample space according to a distribution, which enables global optimization algorithms to be more efficient in determining the lowest-energy configuration of the PES.

### 3.1. Metropolis Sampling

The Metropolis algorithm (Metropolis et al., 1953) has been extensively employed for evaluating thermodynamic properties of a system at some desired temperature. The algorithm begins by initiating a random configurational state (*i*) that is evolved using a Monte Carlo random walk for some pre-defined number of stochastic steps. During the random walk, a new trial configuration (*t*) is generated at each step (by, e.g., the swapping of atomic positions of two different components). If the energy of the random trial state (E_*t*_) is lower than the initial state (*E*_*i*_), the trial state becomes the initial state (*E*_*i*_) in the subsequent stochastic step. If *E*_*t*_ > *E*_*i*_, then the trial state is accepted with the probability of exp(−β(*E*_*t*_ − *E*_*i*_)), where β = 1/*k*_B_*T*. By increasing the temperature, therefore, the acceptance rate of higher-energy trial states is increased compared with sampling at low temperatures.

After a Metropolis run completes at a fixed temperature, properties, such as the average energies of the accepted trial states can be calculated. The entropy and free energy cannot, however, be readily computed from this approach because these statistical properties cannot be expressed as ensemble averages. Instead, thermodynamic integration can be used to estimate the free energy (Tuckerman, 2010). Moreover, one issue with the fixed-temperature sampling of the PES using the metropolis algorithm arises when several low-lying minima exist that are separated by high barriers, which can trap the sampling algorithm locally and limit sampling of the configurational space. Typically, multiple runs are needed to accurately describe quantities over a large range of temperatures (Landau et al., 2004) to avoid the dependence of the results on the starting configuration. See textbooks, such as Landau and Binder (2005) for a more in-depth discussion of Monte Carlo approaches.

### 3.2. Wang-Landau Algorithm

The Wang-Landau (WL) algorithm (Wang and Landau, 2001a,b; Landau and Wang, 2002) allows for the direct estimation the temperature-independent DOS [σ(*E*)] based on an histogram of energies that is updated iteratively as more states are sampled. In the WL algorithm, sampling is performed by initiating a random configurational state that is evolved using a Monte Carlo random walk typically in an energy range *E*_*min*_ < *E* < *E*_*max*_, and the trial structure with energy *E*_*t*_ is accepted based on the probability:

(15) $$\begin{array}{l} \rho \left(E_{i}-E_{t}\right)=\text{min}\left(1,\frac{\sigma \left(E_{\text{i}}\right)}{\sigma \left(E_{\text{t}}\right)}\right), \end{array}$$

where σ(*E*_i_) is the initial DOS and σ(*E*_t_) is the DOS of the trial state. If the trial state is accepted, the DOS histogram is updated according to: σ(*E*_*t*_) = σ(*E*_t_) × *f*, where *f* is the WL factor. If the trial state is rejected, σ(*E*_*i*_) is update by the same factor instead. For the initial iteration, an *f* = *e*^1^ was recommended by Wang and Landau (2001b), which is updated to decrease monotonically in each subsequent iteration.

In addition, a histogram *H*(*E*) corresponding to the frequency a distinct configurational state (*s*) visited during the Monte Carlo random walk is also updated according to:

(16) $$\begin{array}{l} H\left(E_{s}\right)=H\left(E_{s}\right)+1. \end{array}$$

Where *E*_*s*_ = *E*_*t*_ if *E*_*t*_ is accepted, otherwise *E*_*s*_ = *E*_*i*_. *H*(*E*) is initially set it to zero for all *E* because no states have been sampled. The random walk continues until the histogram *H*(*E*) becomes flat for some range of energies and then resets to zero for all energies [in contrast to σ(*E*), which is continuously updated as the calculation progresses]; as *H*(*E*) becomes flatter over larger energy ranges in subsequent iterations, σ(*E*) becomes more accurately estimated. Once σ(*E*) is known, the partition function (see Equation 3) and thermodynamic quantities, such as the internal energy and free energy (see Equation 4) can be estimated at any temperature.

### 3.3. Nested Sampling

The nested sampling (NS) algorithm was originally proposed by J. Skilling for Bayesian computations (Skilling, 2004, 2006) and subsequently adapted for the automated calculation of pressure-temperature composition phase diagrams (Pártay et al., 2010; Baldock et al., 2016, 2017). The key feature of the NS algorithm is the iterative elimination of a fixed fraction of the phase space (corresponding to high-energy subset above some defined energy limit, *E*_*max,i*_), which effectively allows for a top-down energy sweep of phase space and constructing the (cumulative) DOS. At each iteration (*i*) of the NS algorithm, an energy limit (*E*_*max,i*_) is defined and the corresponding state is evolved using a Monte Carlo random walk for some set of pre-defined number of stochastic steps. At each stochastic step, the trial state is accepted if the energy (*E*_*t*_) is below the defined energy limit *E*_*t*_ < *E*_*max,i*_. A new energy limit *E*_*max*_ is selected at each NS iteration and sampling in performed again below this new upper-bound. This sequence of recorded *E*_*max*_ values is the main output from NS and can be viewed as a discretization of the cumulative density of states χ(*E*), which is given by the following:

(17) $$\begin{array}{l} \chi \left(E\right)=\int_{-\infty }^{E}\sigma \left(E^{\prime }\right)dE^{\prime }. \end{array}$$

This is because each energy level *E*_*max,i*_ at iteration *i* can be associated with a fraction of configuration space, where $\chi \left(E_{i}\right)=\alpha^{i}$. The DOS [σ_*i*_(*E*)] is the (normalized) volume of phase space between successive energy levels (i.e., α^*i*^ − α^*i*+1^). This allows for a relatively straight forward calculation of the partition function in Equation (3) *a posteriori* at any value of β = 1/*k*_B_*T* by a discrete sum over the energy levels (ignoring an additional pre-factor due to the momentum):

(18) $$\begin{array}{l} Z\approx \underset{i}{\sum }\left(\alpha^{i}-\alpha^{i+1}\right)e^{-\beta E_{max,i}}. \end{array}$$

With the determination of *Z*, various thermodynamic observables (e.g., internal energy and free energy) can be calculated readily for any temperature.

This iterative (decreasing) constraint on the energy in the nested sampling algorithm allows for an unbiased exploration of the phase space and aims to uniformly sample areas of the phase space where significant changes occur (i.e., during a first-order phase transition, where the available phase space increases as the energy of the system increases). This is in contrast to the WL algorithm, where the phase space is explored by sampling the energy uniformly and could lead to issues with sampling properly near phase transitions (Pártay et al., 2010). A potential limitation of nested sampling algorithm is that this decreasing energy constraint can prevent exploration of an unexplored minima on the PES, which is problematic in dealing with systems exhibiting broken ergodicity (Pártay et al., 2010).

## 4. Cluster Expansion

As discussed in the Introduction, an accurate calculation of the configurational DOS is needed to estimate thermodynamic properties and stability. The CE approach (Sanchez et al., 1984; de Fontaine, 1994; Ducastelle, 1994) provides a numerically efficient way to evaluate the properties of a large number of configurations using a relatively small number of reference calculations in training the model. CE can be combined with stochastic sampling techniques to identify new stable structures and calculate the thermodynamic quantities and phase diagrams. Because of this, CE has become a standard approach for calculating the properties of solid-state alloys (Magri and Zunger, 1991; Asta et al., 2001; Franceschetti et al., 2006; Ruban and Abrikosov, 2008; Casola et al., 2010; Chan et al., 2010; Wu et al., 2016), to identify key arrangements of surfaces (Borg et al., 2005; Cao et al., 2018) and adsorbate layers (Stampfl et al., 1999), and modeling materials with vacancies or defects (Van der Ven et al., 2001; Van der Ven and Ceder, 2005; Muzyk et al., 2011; Zhang and Sluiter, 2015). Several CE packages are available including the Universal Cluster Expansion Code (UNCLE) (Lerch et al., 2009), Alloy Theoretic Automated Toolkit (ATAT) (van de Walle et al., 2002), Clusters Approach to Statistical Mechanics (CASM) software (CASM, 2017), CLUPAN (Seko et al., 2009), CLEASE (Chang et al., 2019), and Cluster Expansion for large parent ceLLs (CELL) (Troppenz et al., 2017). In section 5.1, we will demonstrate how CE can be used to evaluate the energy of a large number of configurations to model thermodynamic properties of an exemplary binary alloy. Here, we provide the necessary background of the CE method (Sanchez et al., 1984; de Fontaine, 1994; Ducastelle, 1994).

To illustrate how the CE model works, consider a simple binary alloy *A*_*x*_*B*_1−*x*_ with only two atomic species (*A* and *B*). The CE model relies on the fact that a crystalline material with *N* atomic sites can be represented as a *N*-dimensional vector $\vec{\eta }$ of the occupation of each atomic site *i* (1, ..., *N*):

(19) $$\begin{array}{l} \vec{\eta }=\left(\eta_{1},...,\eta_{\text{N}}\right) \end{array}$$

where η specifies which type of atom occupies a given site, e.g., η = +1 (η = −1) if the site is occupied by atom *A* (atom *B*).

The energy of the configuration, $E\left(\vec{\eta }\right)$ (or any other configuration-dependent property), can then be modeled as a function of $\vec{\eta }$:

(20) $$\begin{array}{l} E\approx E\left(\eta_{1},...,\eta_{\text{N}}\right) \end{array}$$

$E\left(\vec{\eta }\right)$ can be represented in an orthonormal basis set of *clusters* (α). Different choices of the cluster basis functions have been implemented (Sanchez et al., 1984; van de Walle, 2009), consisting of combinations of lattice sites α = (*i, j, k*, ...) up to the *N*-site cluster, which represent the different types of interactions:

(21) $$\begin{array}{l} E_{s}\left(\vec{\eta }\right)=E_{s}^{\text{CE}}\left(\vec{\eta }\right)=\underset{\alpha }{\sum }\upsilon_{\alpha }J_{\alpha }X_{s\alpha }, \end{array}$$

where *J*_α_ is the regression coefficient associated with a cluster α and the sum runs over all possible inequivalent clusters. The multiplicity υ_α_ accounts for clusters that are symmetrically equivalent to α. The value *X*_*sα*_ represents the correlation of the cluster α with the configuration $\vec{\eta_{s}}$ and is calculated by taking the products of the occupation value η_*i*_ for each site:

(22) $$\begin{array}{l} X_{s\alpha }=\frac{1}{\upsilon_{\alpha }}\underset{\alpha^{\prime }\equiv \alpha }{\sum }\underset{i\in \alpha^{\prime }}{\prod }\eta_{si}. \end{array}$$

The sum runs over the set of clusters (α′) that are symmetrically equivalent to α. The product of the occupation variables η_*si*_ runs over all lattice sites *i*. This operation is performed for each configuration *s*, which leads to structure-specific values that depend on the lattice site occupations. The values for *X*_*sα*_ range between −1 and +1 and are essentially an average of the rotated/translated clusters used in the model over the occupation of each lattice site when a binary cluster function is used.

**Figure 2:**
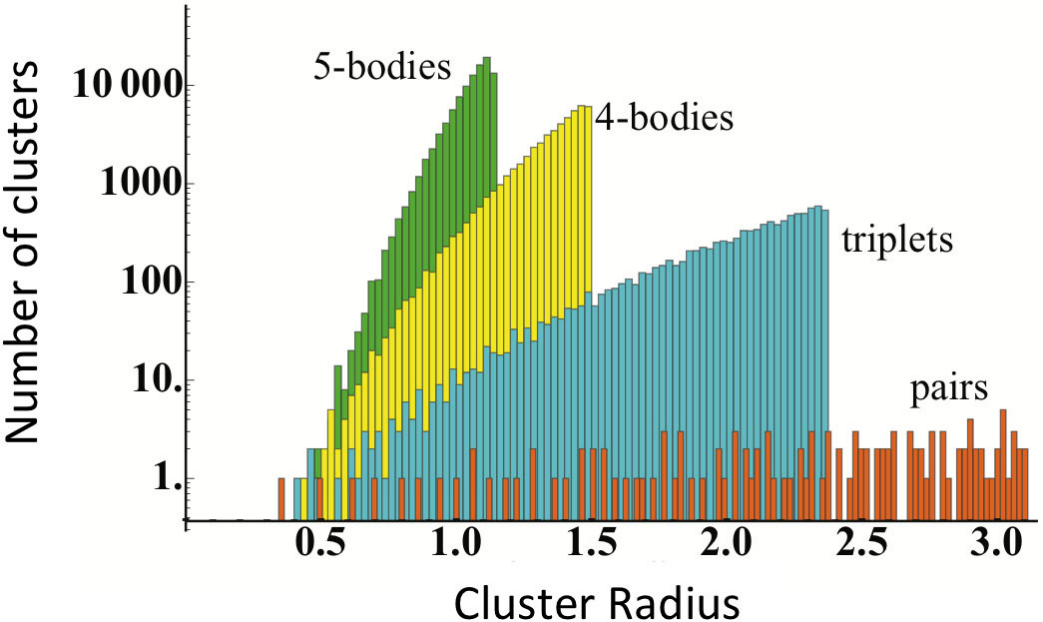
Number of clusters as a function of the cluster radius in units of lattice constant for an *fcc* system. Reproduced from Nelson et al. (2013) with permission from APS.

Obtaining an accurate CE model requires determining the optimal *J*_α_ values. The set of possible clusters are practically infinite, however, which is underscored for an *fcc* system in Figure 2. Typically, this is solved by taking advantage of the “nearsightedness” of interactions in the solid-state by truncating the set of clusters. For example, by only including only one-, two-, and three-body clusters within a relatively small cutoff. If the number of clusters is smaller than the number of DFT calculations used for training, it is straightforward to use linear regression to determine the *J*_α_ values.

As an alternative approach, the training of a cluster expansion model can be represented as a minimization of equation:

(23) $$\begin{array}{l} loss=\frac{1}{N}\underset{s}{\sum }{\left(E_{s}^{\text{CE}}-E_{s}\right)}^{2}+\lambda \underset{\alpha }{\sum }|J_{\alpha }{|}^{l}\text{.} \end{array}$$

The first term (i.e., the mean-squared error) ensures that the cluster expansion model has a low overall error, which is penalized by the second term that is weighted by the regularization parameter λ. This penalty term is used because an additional goal is to select only the best and smallest subset out of the large set of possible clusters. This is referred to as a “sparse” solution because there are only a few non-zero components compared with the total number of possible clusters. To optimize a sparse solution using Equation (23), a few values of *l* can be used. If *l* = 0 (the so-called *l*_0_ norm), then the mean squared error is penalized by the total number of non-zero *J*_α_ values. However, Equation (23) with *l* = 0 is challenging to solve because there is a combinatorially large number of possible solutions. Instead, *l* = 1 (the so-called *l*_1_ norm) is commonly used in Equation (23), which allows for a convex minimization problem whose solution is the same or close to the optimal solution with the *l*_0_ norm. The *l*_1_ norm optimization is typically used in the field of compressed sensing but has recently been adapted to training CE models in one shot (Nelson et al., 2013).

It should be noted that the optimal regularization parameter λ in Equation (23) is selected from a set of values within some pre-defined range and step-size. The total CE model (i.e., set of α and corresponding *J*_α_ and λ) should be optimized via Equation (23) to ensure the predictive accuracy is preserved on data outside of the training set. This is accomplished by evaluating the model error on data that was withheld from the model training (i.e., a test set). Because the solution could therefore depend on which samples are in the training and test set, a common strategy to reduce the variance in the estimated error across splits is to use cross-validation (*CV*), which involves partitioning the dataset into several non-overlapping training/test splits. For example, in leave-one-out cross validation (*CV*_*LOO*_), *k*-folds are created (where *k* is the number of structures in the training set) each containing only one structure in the test set. The CE model is trained on *k*-1 structures, and the error is calculated for this single excluded structure and averaged over all *k*-folds:

(24) $$\begin{array}{l} CV_{LOO}=\frac{1}{k}\underset{s}{\sum }{\left(E_{s}^{\text{CE,}k-1}-E_{s}\right)}^{2}, \end{array}$$

where *s* is the withheld structure and $E_{s}^{\text{CE},k-1}$ is the energy of structures using the optimal CE model parameters (both the optimal penalization strength λ and the optimal set of *J*_α_ values) obtained by minimizing the error for *k*-1 structures. The *CV* scheme has been widely employed to build accurate CE models because it helps ensure that the predictive accuracy of the CE model obtained for relatively small number of reference calculations can generalize to structures outside of the training set (van de Walle et al., 2002).

Although the optimal choice of clusters and *J*_α_ values is crucial for obtaining an accurate model, we note that the accuracy of CE is typically limited in cases where large geometry relaxations away from ideal lattice positions occur. This is because of an inherent limitation in the representation of a complex geometry by a vector of lattice site occupations via Equation (19).

## 5. Examples

### 5.1. Thermodynamics of Alloys

In this section, we demonstrate the main concepts of thermodynamics of alloys in an examination of the Cu-Au binary alloy, which provides a convenient system for analysis because it has been investigated in several previous studies (Ozoli et al., 1998; Wolverton et al., 1998).

At *T* = 0 K, the stability of a material can be estimated if its formation energy per atom [*E*(*x*)] is lower than all other compounds at all relevant compositions. This is determined by identifying the set of compounds that form the convex hull, which is the convex boundary of the 2D landscape of energies and compositions.

**Figure 3:**
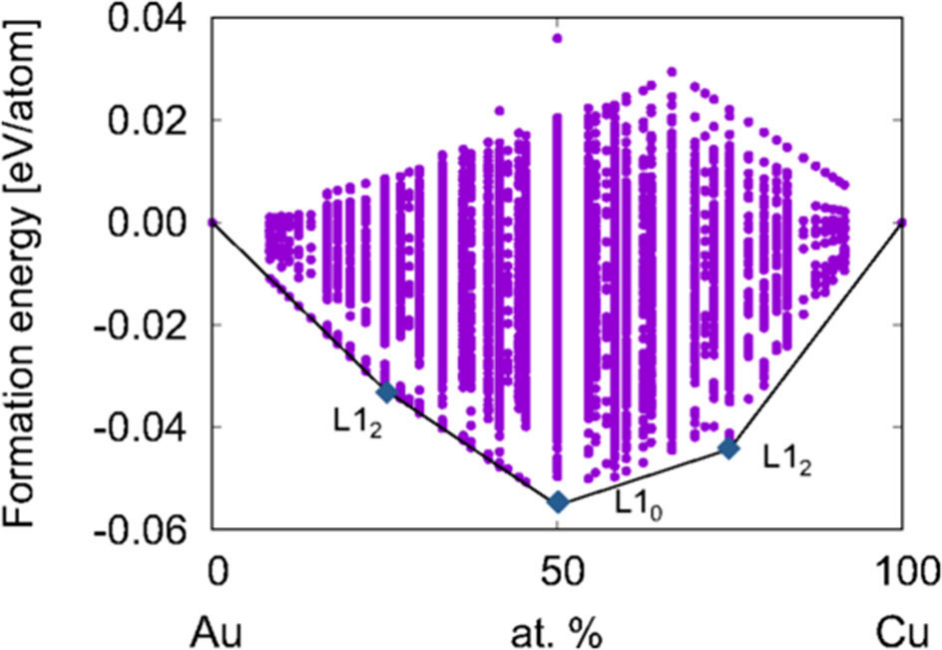
Convex hull of Cu_*x*_Au_1−*x*_. Reproduced from Takeuchi et al. (2017) with permission from APS.

The convex hull for ca. 10,000 Cu_*x*_Au_1−*x*_ structures is shown in Figure 3. *E*(*x*) is computed by subtracting the energies of the pure metals (using the energy per atom of Au [*E*(Au)] and Cu [*E*(Cu)] in the *fcc* lattice):

(25) $$\begin{array}{l} \Delta E\left(x\right)=E\left({\text{Cu}}_{x}{\text{Au}}_{1-x}\right)-xE\left(\text{Cu}\right)-\left(1-x\right)E\left(\text{Au}\right). \end{array}$$

The points above the convex hull correspond to higher-energy configurations states that are considered to be unstable because the system can minimize the total energy by relaxing to a lower energy structure either at that composition or into other compositions. Accurately modeling the stability therefore requires inputting the correct set of configurations into the convex hull construction.

**Figure 4:**
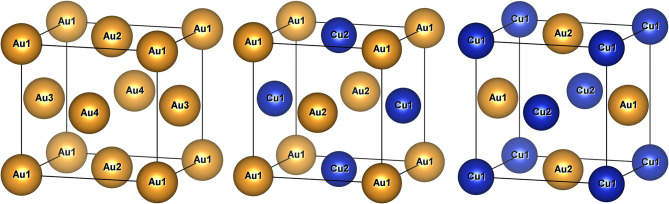
Illustration of the unsubstituted Au and two CuAu configurations in the (*L*1_0_ distorted) *fcc* lattice, which has four inequivalent lattice positions. The equivalent lattice sites by translational symmetry are labeled accordingly.

It can be observed from Figure 3 that finding the lowest-energy configuration at a given composition is computationally difficult because of the large number of states that have to be analyzed. Indeed, for a binary mixture, there are ~2^*N*^ ways to decorate the lattice, where *N* is the number of possible lattice sites. Some of the resulting 2^*N*^ configurations may be equivalent due to symmetry, but the scaling with *N* remains exponential, leading to a large configurational space. As an example, three configurations are illustrated in Figure 4 for CuAu in the *L*1_0_ distorted *fcc* lattice.

**Figure 5:**
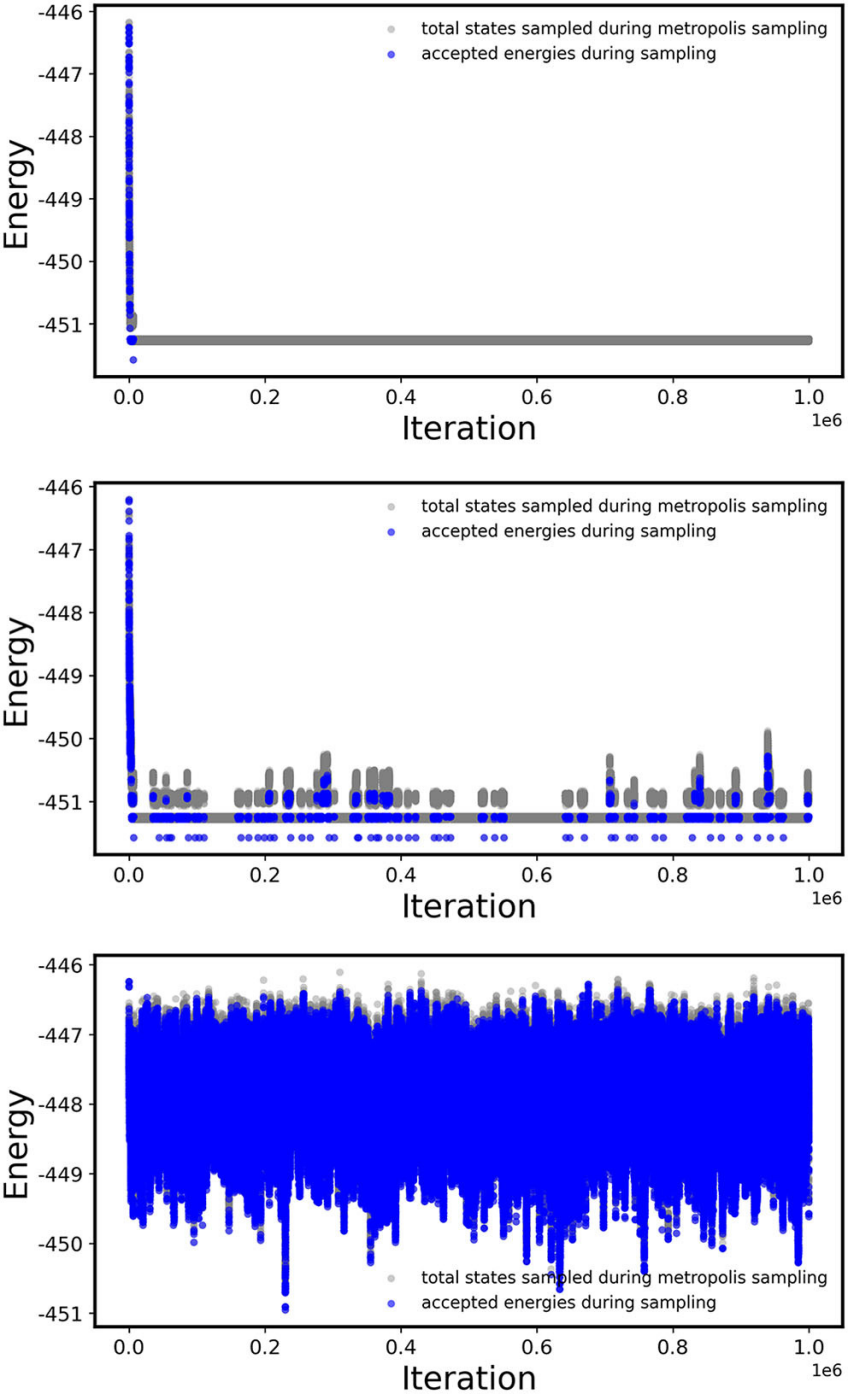
Trial (gray) and accepted (blue) configuration energies (in eV) at each iteration during a single Metropolis run at *T* = 200 K (top), *T* = 400 K (middle), and *T* = 800 K (bottom).

The CE model provides an efficient method for evaluating the energy of each configuration, which can be combined with stochastic sampling techniques for efficiently searching a large configurational space. This is demonstrated for one composition, CuAu, using the Metropolis algorithm for three different temperatures in Figure 5. In this illustrative example, the CE model consists of up to two-body clusters within a radius of 6 Å and was obtained with *CV*_*LOO*_ on a previously computed training set of 32 structures taken from Chang et al. (2019).

**Figure 6:**
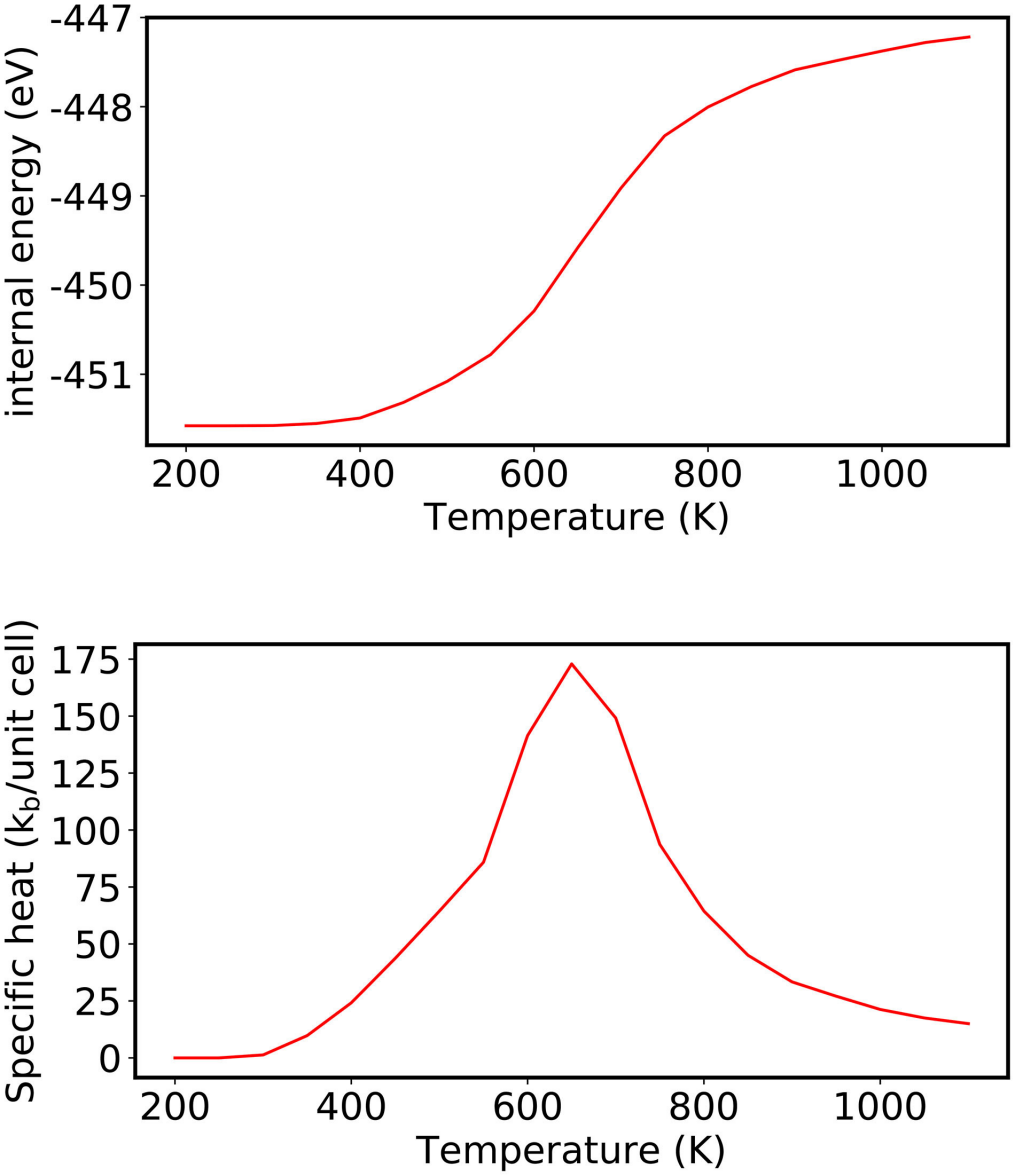
(Top) Evolution of the internal energy of CuAu and (Bottom) corresponding heat capacity, where the peak corresponds to the order-disorder transition determined using the Metropolis algorithm for the range of temperatures from 200 to 1,100 K, evaluated in 50 K increments.

Using the Metropolis algorithm, each trial configurations are accepted with the probability exp(−β(*E*_*t*_ − *E*_*i*_)), leading to the sampling of higher-energy trial configurations at higher temperatures. This is evident in Figure 5 where at the *T* = 200 K only the lowest-energy ordered structure is adopted and only one other higher-energy trial state is generated (but not accepted). In contrast, at *T* = 800 K, several configurational states are accepted, effectively leading to the case where the atomic species are randomly distributed over the various lattice sites. The larger range of energies sampled with increasing temperature in turn increases the internal energy of the system, which is estimated as the average 〈*E*〉_*T*_ of the configurational states sampled with the probability exp(−β(*E*_*t*_ − *E*_*i*_)) at a given temperature. (Note: for an unbiased averaging, the energies are taken only after some pre-defined initial set of stochastic steps are performed, which would exclude the initial almost linear decrease in the accepted trial energy.) The temperature where the systems transitions from a predominately ordered structure to the completely disordered state is indicated by a non-linear change in the internal energy of the system beginning around ca. *T* = 600 K. The temperature of the phase transition is observed more clearly as a peak in the specific heat (*C*_*v*_) in the bottom panel of Figure 6, which is calculated (in units of *k*_*B*_) from the following equation (Frenkel and Smit, 2001):

(26) $$\begin{array}{l} C_{v}\left(\beta \right)=\beta^{2}\left({\langle E^{2}\rangle }_{T}-{\langle E\rangle }_{T}^{2}\right). \end{array}$$

Using Equation (26), the specific heat reaches a peak at *T* = 650 K for CuAu, which is comparable values previously reported using CE combined with metropolis algorithm (Ozoli et al., 1998; Wolverton et al., 1998; Chang et al., 2019) and experiment [*T* = 680 K (Okamoto et al., 1987)].

For comparison, the order-to-disorder transition temperature is also computed for CuAu using the NS algorithm (using 100 random walkers, 4,000 NS iterations to construct the set of maximum energies *E*_*i*_, and 40 stochastic steps were used to randomize the walkers at each NS iteration, *i*). As discussed in section 3.3, all thermodynamic properties can be extracted from the list of successive energy levels, which are used to determine the DOS. For example, the evolution of the average energy of the system with increasing temperature [*U*(β)] can be evaluated:

(27) $$\begin{array}{l} U\left(\beta \right)=\frac{1}{Z\left(\beta \right)}\underset{i}{\sum }\left(\alpha^{i}-\alpha^{i+1}\right)E_{i}e^{-\beta E_{i}}, \end{array}$$

The heat capacity using NS can then be evaluated according to the following equation:

(28) $$\begin{array}{l} C_{v}\left(\beta \right)=\beta^{2}\left(\frac{1}{Z\left(\beta \right)}\underset{i}{\sum }\left(\alpha^{i}-\alpha^{i+1}\right)E_{i}^{2}e^{-\beta E_{i}}-U{\left(\beta \right)}^{2}\right), \end{array}$$

where β = 1/*k*_*B*_*T*.

**Figure 7:**
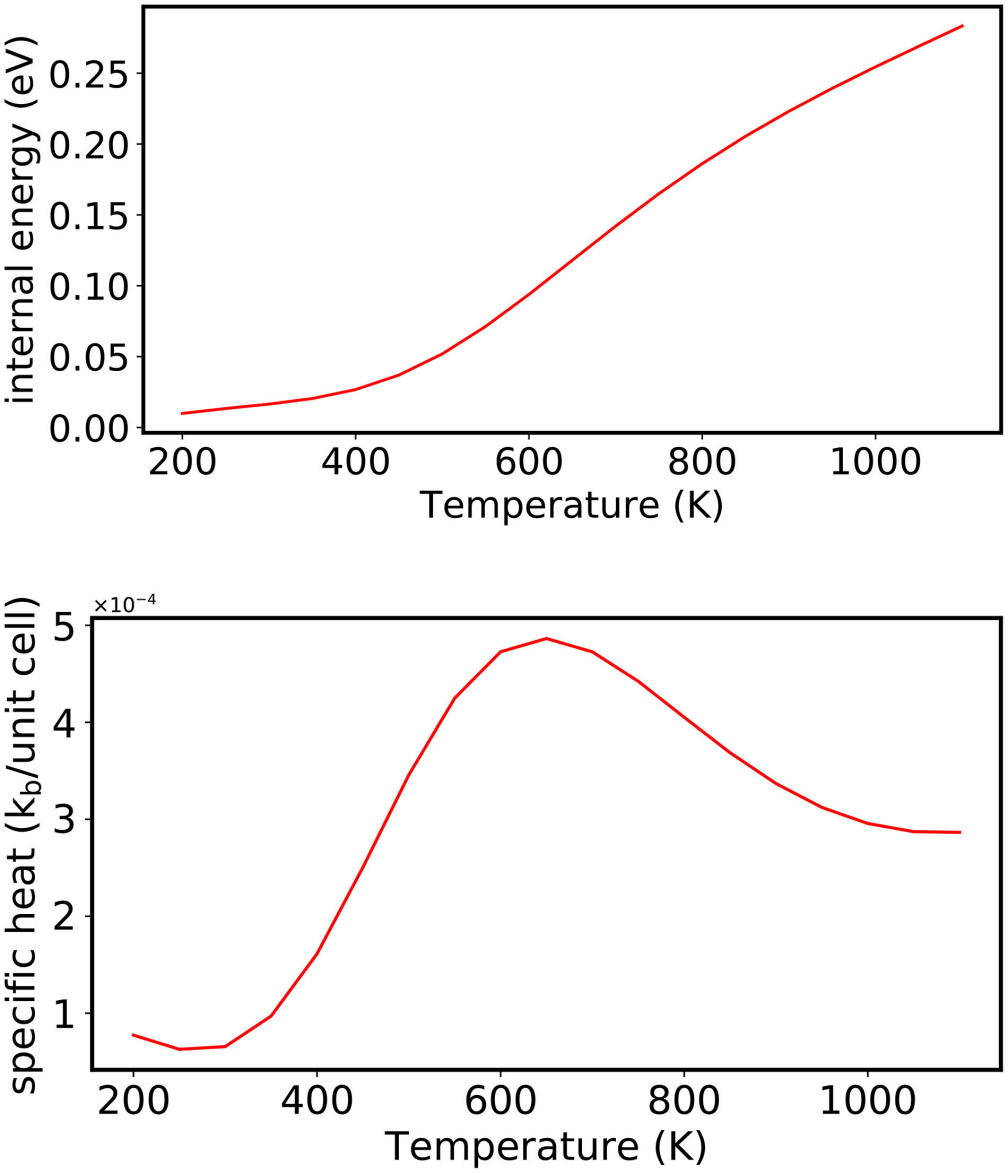
(Top) Evolution of the average energy of the system with increasing temperature (in 10 K increments) analyzed as a post-processing scheme from NS. (Bottom) An order disorder transition is observed by the largest change in the internal energy, which is indicated by a peak in the heat capacity at *T* = 645 K. The energies in the plot were shifted by the minimum outer energy entry.

Using NS, the order-disorder phase transition at *T* = 645 K is determined from the maximum in the heat capacity shown in the bottom panel of Figure 7, which compares well with what is obtained using the Metropolis algorithm for the same CE model and experiment.

### 5.2. Point Defect Concentrations

Point defects, such as vacancies, interstitials, antisite defects, and substitutional defects, can form spontaneously or intentionally in a material. For example, the bright colors observed in precious stones, such as ruby and sapphire are due to point defects (different defects and concentrations resulting in strikingly different colors). A very small concentration of impurities in Si (intentionally introduced at the level one per billion Si atoms) determines its semiconducting properties that are the basis of almost all modern electronics. Charge transport, thermoelectric and optical properties of materials are often determined by the presence and distribution of defects. The ability to predict the behavior of point defects at realistic temperatures and pressures is therefore very important (Van der Ven et al., 2001; Van der Ven and Ceder, 2005; Osorio-Guillén et al., 2007; Muzyk et al., 2011; Gopal and van de Walle, 2012; Zhang and Sluiter, 2015).

The formalism presented in this section can be found in Drabold and Estreicher (2007); here, we just give a general overview. Let us first consider the simplest example of non-interacting point defects in a crystal. This is usually a sufficiently good model for charge-neutral defects, provided their concentration is not too high, and there is no strong short-range attractive interaction between the defects. This is the case, e.g., for oxygen vacancies in MgO. For simplicity, let us also focus on the case of a single defect site type, i.e., all possible crystal lattice sites where a defect can form are equivalent. For non-interacting defects, the formalism presented below is trivially extended to the case of multiple site types by treating defects in each non-equivalent site of the sublattice independently.

At fixed (*T, p*) conditions, the system will tend to the minimum of its Gibbs free energy with respect to the number of defects *N*. The free energy can be written as follows:

(29) $$\begin{array}{l} G\left(T,p,N\right)=G^{\text{perf}}\left(T,p\right)+N\Delta G_{\text{f}}\left(T,p\right)-TS_{\text{conf}}\left(N\right), \end{array}$$

where *G*^perf^ is the Gibbs free energy of the system without defects, *S*_conf_ is the configurational entropy, and Δ*G*_f_ is the Gibbs free energy of the defect formation:

(30) $$\begin{array}{l} \Delta G_{\text{f}}\left(T,p\right)=G^{\text{def}}\left(T,p\right)-G^{\text{perf}}\left(T,p\right)-\underset{i}{\sum }\Delta n_{i}\mu_{i}\left(T,p_{i}\right). \end{array}$$

Here, *G*^def^ is the Gibbs free energy of the system with a single defect, Δ*n*_*i*_ are the changes in the number of atoms of type *i* with respect to the perfect system for a single defect that is either removed (i.e., a vacancy, Δ*n* = −1) or added (i.e., an interstitial, Δ*n* = 1), and μ_*i*_(*T, p*_*i*_) are chemical potentials as defined in Equation (5). Note that Δ*G*_f_ contains all entropic contributions (vibrational, electronic, etc.) except the configurational entropy. In thermodynamic equilibrium, defects with a positive formation energy are stabilized by their configurational entropy, which was previously shown to be the largest contribution to the overall entropy (Estreicher et al., 2004). Recent results suggest, however, that vibrational contributions to the defect formation energies are also important (Sun et al., 2018), but are often neglected because of high computational cost (Fultz, 2010). These results suggest the need to investigate these contributions for each specific material of interest.

In order to minimize Equation (29), *S*_conf_ as a function of *N* has to be determined. For non-interacting defects, the configurational DOS, and, consequently, the partition function, can be trivially calculated, since all configurations have the same energy: the DOS is equal to the number of non-equivalent ways by which *N* defects can be distributed among *N*_sites_ (multiplied by the Dirac δ-function centered at the total energy *E*(*N*) of the system with N defects). This number is the binomial coefficient $C_{N_{\text{sites}}}^{N}=N_{\text{sites}}!/N!\left(N_{\text{sites}}-N\right)!$. In addition, each defect may have its own internal configurational freedom, e.g., due to the crystal symmetry. The partition function for a given *N* is thus

(31) $$\begin{array}{l} Z\left(N\right)=\Omega^{N}C_{N_{\text{sites}}}^{N}e^{-E\left(N\right)/k_{\text{B}}T}, \end{array}$$

where Ω is the on-site configurational degeneracy. Using Equation (4), we obtain the configurational entropy:

(32) $$\begin{array}{l} S_{\text{conf}}\left(N\right)=k_{\text{B}}\text{ ln }\Omega^{N}C_{N_{\text{sites}}}^{N}. \end{array}$$

To minimize *G*(*T, p, N*) from Equation (29), one can calculate its derivative with respect to *N* and set it to zero. For this, we need to cast Equation (32) into a differentiable form. For a macroscopic system with a large number of defects and defect sites (≥ 10^10^), Stirling's formula (ln *N*! ≈ *N*ln *N* − *N*) gives a very good differentiable approximation of a factorial. Using the formula and minimizing Equation (29) with respect to *N*, we obtain the following:

(33) $$\begin{array}{l} N=N_{\text{sites}}\Omega /\left(\text{exp}\left(\Delta G_{\text{f}}/k_{\text{B}}T\right)+\Omega \right). \end{array}$$

In textbooks, often an Ω = 1 is assumed. Also, Δ*G*_f_/*k*_B_*T* ≫ 1 is typically assumed, which corresponds to a small concentration of defects, *N* ≪ *N*_sites_. In this case, the concentration is

(34) $$\begin{array}{l} N\approx N_{\text{sites}}\text{exp}\left(-\Delta G_{\text{f}}/k_{\text{B}}T\right). \end{array}$$

This is a reasonable approximation when the defect formation energy is large compared to thermal energy *k*_B_*T*, which is a typical situation for stable materials. This approximation is also consistent with the assumption that defects do not interact because at small concentrations the average distance between defects is large. However, there are practical situations when *N* ~ *N*_sites_, but Equation (33) is still applicable, namely, when the concentration of defect sites themselves is small. In this case, a situation can occur when several types of defects compete for available sites, but the interaction between them can still be neglected [e.g., for defects at corners of the MgO surface (Bhattacharya et al., 2017)]. A naive application of Equation (33) to determine concentration of each type of defect independently fails because it could yield a non-physical total concentration of defects greater than the concentration of possible defect sites. Instead, we have to take into account the fact that the number of available sites for each defect type is reduced, resulting in a coupled system of equations:

(35) $$\begin{array}{l} N_{i}=\left(N_{\text{sites}}-\underset{j\neq i}{\sum }N_{j}\right)\Omega_{i}/\left(\text{exp}\left(\Delta G_{\text{f}}^{\left(i\right)}/k_{\text{B}}T\right)+\Omega_{i}\right), \end{array}$$

with index *i* labeling defect types. Equation (35) is easily solved to give:

(36) $$\begin{array}{l} N_{i}=N_{\text{sites}}\frac{\Omega_{i}e^{-\Delta G_{\text{f}}^{\left(i\right)}/k_{\text{B}}T}}{\left(\sum_{j}\Omega_{j}e^{-\Delta G_{\text{f}}^{\left(j\right)}/k_{\text{B}}T}+1\right)}. \end{array}$$

Now, $\underset{i}{\sum }N_{i}<N_{\text{sites}}$ is always fulfilled.

In the case of a short-ranged (see below) interaction between defects, similar considerations can be applied to determine the concentration of the defects and their clusters. Let us consider a fixed total number *N* of dopant atoms *A* in a material (e.g., a non-volatile transition-metal impurity in an oxide). If the dopants interact with an attractive potential that decays with distance, we can interpret every unique (not convertible to one another by symmetry operations) combination of 1, 2, etc. dopants as a distinct defect type *i*. Since *N* is fixed, the chemical potential of species *A* is not fixed in this case, it is determined by the final concentrations of the defect clusters (including size 1, which corresponds to a single dopant). If we assume that the concentration of the dopants is small, so that when they are evenly distributed in the system the distance between them is much larger than the interaction length scale, we can write Equation (29) as the following:

(37) $$\begin{array}{l} G\left(T,p,\left\{N_{i}\right\}\right)=G^{\text{ref}}\left(T,p,N\right)+\underset{i}{\sum }N_{i}\Delta I^{\left(i\right)}\left(T,p\right)-TS_{\text{conf}}\left(\left\{N_{i}\right\}\right), \end{array}$$

where *G*^ref^ is the free energy of the reference system where all *A* are far from each other. $\Delta I_{\text{f}}^{\left(i\right)}\left(T,p\right)$ are interaction free energies:

(38) $$\begin{array}{l} \Delta I^{\left(i\right)}\left(T,p\right)=\Delta G_{\text{f}}^{\left(i\right)}\left(T,p\right)-m_{i}\Delta G_{\text{f}}^{\left(1\right)}\left(T,p\right), \end{array}$$

where $\Delta G_{\text{f}}^{\left(i\right)}$ is the formation energy of a dopant cluster of type *i* containing *m*_*i*_ atoms *A*, and $\Delta G_{\text{f}}^{\left(1\right)}$ is the formation energy of the defect containing a single atom *A*. Note that μ_*i*_ in $\Delta G_{\text{f}}^{\left(i\right)}$ are dummy variables and can be chosen arbitrarily in this case, since Δ*I*^(*i*)^ does not depend on μ. Following the same logic as for Equation (35), i.e., each cluster type competes for its sites, and taking into account the conservation of the number of *A*,

(39) $$\begin{array}{l} \underset{i}{\sum }N_{i}m_{i}=N, \end{array}$$

we obtain for the concentrations:

(40) $$\begin{array}{l} N_{i}=N\Omega_{i}\frac{\text{exp}\left(-\Delta I^{\left(i\right)}/k_{\text{B}}T\right)}{\sum_{j}m_{j}\Omega_{j}\text{exp}\left(-\Delta I^{\left(j\right)}/k_{\text{B}}T\right)}. \end{array}$$

One should remember that this formula was obtained assuming small concentrations of *A*. For larger concentrations, unlimited cluster size, and for long-range interactions (like the Coulomb interaction), a combination of the CE method introduced in section 4 and Monte Carlo sampling can be used to calculate the configurational DOS and cluster concentrations (Van der Ven et al., 2001; Van der Ven and Ceder, 2005; Osorio-Guillén et al., 2007; Muzyk et al., 2011; Gopal and van de Walle, 2012; Zhang and Sluiter, 2015).

Defects can be charged by losing or acquiring electrons, which can significantly alter their properties. The formation energy of an isolated charged defect can be calculated using Equation (30), where one of the particle types is electron, and the corresponding chemical potential μ_e_ is the Fermi level. In thermodynamic limit (number of particles in the system approaching infinity) any finite concentration of charge would result in an infinitely high energy. This is the consequence of the long-range nature of the Coulomb interaction. Charged defects must therefore be compensated either by defects of opposite charge or by delocalized electrons or holes so that the system remains overall neutral. This charge neutrality condition determines the electronic chemical potential of a system with charged defects, which implies that charged defects cannot be considered non-interacting. This interaction results in a non-trivial distribution of the configurational DOS, which can be examined through sampling. In the case of localized charged defects, however, the long-range part of the interaction can be sampled with a simple electrostatic model, where each defect is represented by multipoles interacting with other defects by a screened Coulomb interaction (depending on the static dielectric tensor of the solid). So far the most common approach in the literature is to use an even a simpler model, where the Coulomb interaction between defects is completely neglected, but the charge neutrality condition is still enforced and determines μ_e_ (Freysoldt et al., 2014). This in turn affects the defect formation energies and consequently their concentrations (Freysoldt et al., 2014):

(41) $$\begin{array}{l} N_{i}\left(\mu_{\text{e}}\right)\approx N_{\text{sites}}e^{-\Delta G_{\text{f}}^{\left(i\right)}\left(\mu_{\text{e}}\right)/k_{\text{B}}T},\underset{i}{\sum }N_{i}\left(\mu_{\text{e}}\right)q_{i}=0, \end{array}$$

where *q*_*i*_ are the defect charges. Physically, this approximation corresponds to a very low concentration of charged defects. The formation energy should be calculated in this case for isolated charged defects. In periodic models, there is an artificial interaction between the defect and the compensating background charge, as well as between the defect and its images in other unit cells. This interaction affects the calculated formation energy and must be removed (Makov and Payne, 1995; Freysoldt et al., 2009; Komsa and Pasquarello, 2013).

In the case of compensation by delocalized charge carriers, the overall Coulomb interaction energy will depend on the electronic DOS (number of electronic states per energy unit per volume unit) near the Fermi level. This opens up an intriguing possibility of tuning defect concentrations by modifying the charge-carrier dopant distribution (doping profile), which determines the electronic DOS at the Fermi level. Formation of charged defects at surfaces of semiconductors is accompanied by formation of a space-charge layer under the surface, and the energy of the induced electric field can be a significant part of the energy of the whole system (Richter et al., 2013). In general, charge-carrier doping is ubiquitous and should be treated as a variable defining thermodynamic equilibrium along with temperature and pressure.

### 5.3. Surface Phase Diagrams

A surface of a solid material under a gaseous atmosphere is constantly bombarded by the molecules or atoms of the gas. Assuming that the gas can be described approximately as an ideal gas, we can estimate the molecular flux ν, i.e., the number of molecules (or atoms for an atomic gas) hitting the surface per unit area per second (Reif, 2000):

(42) $$\begin{array}{l} \nu =\frac{p}{\sqrt{2\pi mk_{\text{B}}T}}, \end{array}$$

where *p* is the pressure of the gas, *m* is the mass of the molecule/atom, and *T* is the temperature. In, for example, an oxygen atmosphere (*m* ≈ 2.66 · 10^−26^ kg) at *T* = 300 K and *p* = 1 atm, about 4 · 10^6^ molecules thus hit each square Angstrom of the surface per second. This implies that at realistic *T* and *p* the thermodynamic equilibrium between the surface and the gas can be reached relatively quickly. At low pressures or/and high temperatures, the surface may also lose atoms to the gas phase, until the equilibrium with the gas phase is restored. The equilibrium structure and composition of the surface will therefore follow the changes in temperature and pressure and may be very different from the bulk composition.

Let us consider a Pd(100) surface in an O_2_ atmosphere as an example (Todorova et al., 2003; Reuter and Scheffler, 2004). In order to find the equilibrium composition and structure of the surface at a given *T* and *p*, we have to minimize the Gibbs free energy of the surface with respect to the number of O atoms at these fixed *T* and *p*. In general, this implies that we also have to find energies of all possible configurations of the *N*_O_ O atoms at the surface and in the subsurface layers of the system, and all possible surface reconstructions, and calculate the free energy including the configurational entropy from the partition function. Depending on the material, a whole ensemble of (quasi-)degenerate structures may co-exist simultaneously in different parts of the surface. In practice, a simplified approach is often taken by considering a limited number of configurations, constrained by periodic boundary conditions.

A slab model is commonly used in DFT calculations to describe surfaces of a crystal (Sholl and Steckel, 2009). Based on Equation (9), the surface free energy is defined:

(43) $$\begin{array}{l} \gamma \left(T,p,N_{\text{O}}\right)=\frac{1}{A}\left(G_{\text{slab}}\left(T,p,N_{\text{O}}\right)-N_{\text{O}}\mu_{\text{O}}\left(T,p\right)-N_{\text{Pd}}g_{\text{Pd}}^{\text{bulk}}\left(T,p\right)\right), \end{array}$$

where *G*_slab_ is the free energy of the slab model per unit cell, *A* is the area of the surface unit cell, *N*_O_ and *N*_Pd_ are the numbers of O and Pd atoms per unit cell, respectively, and $g_{\text{Pd}}^{\text{bulk}}$ is the free energy of Pd bulk crystal per atom. Note that γ is the sum of free energies of the two surfaces of the slab. In principle, the energy of each surface can be calculated separately by choosing a slab model with two identical surfaces and dividing γ by 2. In practice, however, this may require a large thickness of the slab model, to avoid the artificial interaction between the two surfaces of the slab. Also, choosing equivalent surfaces on the two sides of the slab is not always possible, namely when the inversion symmetry is broken along the surface normal (Levchenko and Rappe, 2008). Fortunately, for constructing a surface phase diagram only knowledge of the relative free energy Δγ with respect to a particular surface state is necessary. For example, we can choose a clean Pd(100) slab as the reference:

(44) $$\begin{array}{l} \Delta \gamma \left(T,p,N_{\text{O}}\right)=\frac{1}{A}\left(\Delta G_{\text{slab}}\left(T,p,N_{\text{O}}\right)-N_{\text{O}}\Delta \mu_{\text{O}}\left(T,p\right)\right), \end{array}$$

where Δ*G*_slab_(*T, p, N*_O_) = *G*_slab_(*T, p, N*_O_) − *G*_slab_(*T, p*, 0) − *N*_O_*E*_O_2__/2, and Δμ_O_ = μ_O_ − *E*_O_2__/2. We emphasize that, although Δ*G*_slab_ is expected to converge fast with slab thickness, this convergence must be carefully tested.

**Figure 8:**
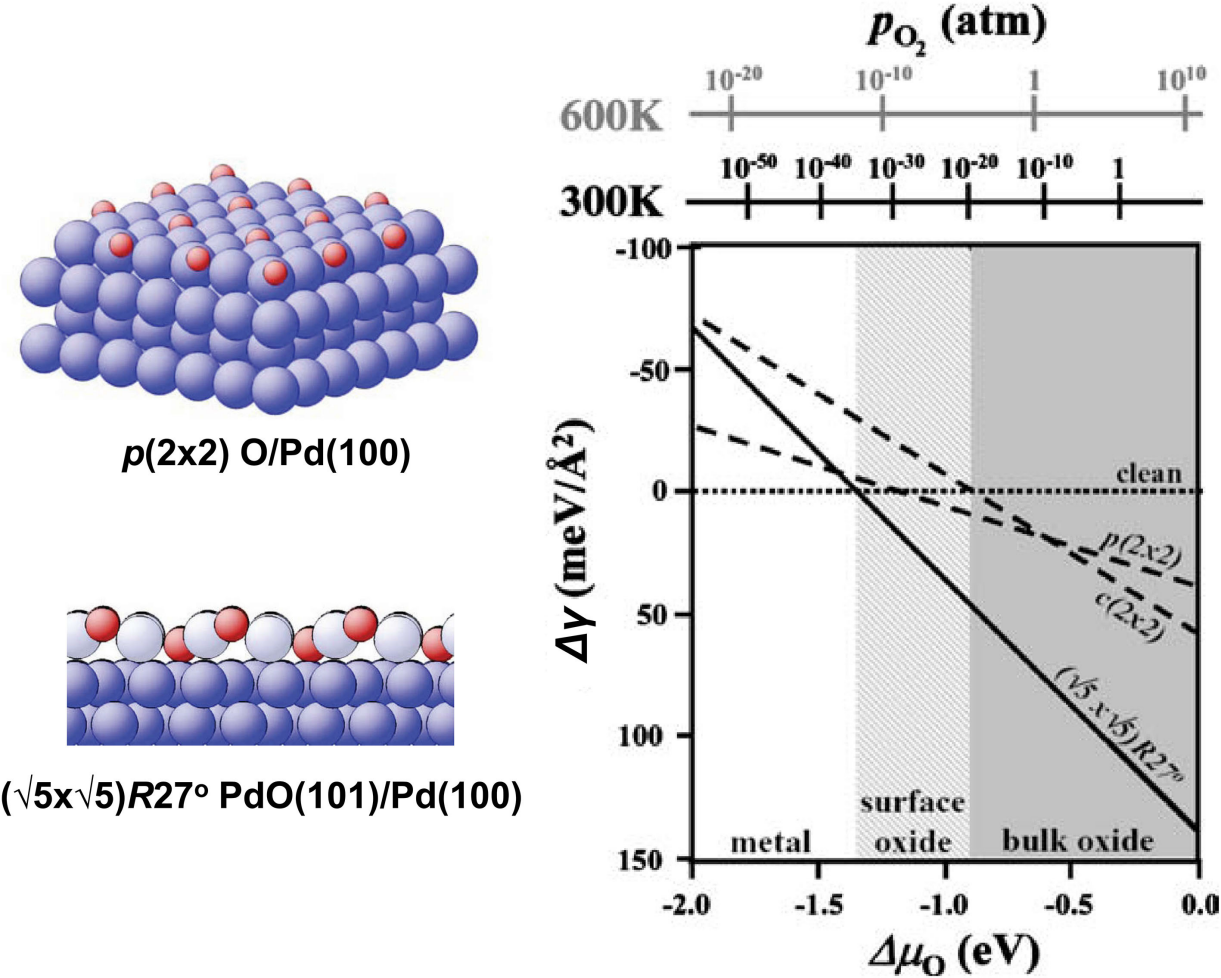
Surface phase diagram for Pd(100) in an O_2_ atmosphere. Reproduced from Reuter and Scheffler (2004) with permission from Springer.

As pointed out in section 2, the *p*Δ*V* contribution to the relative free energy is usually very small and can be neglected (Reuter and Scheffler, 2001). For now, we will also neglect the vibrational and configurational entropy contributions (their effects will be discussed later; see Borg et al., 2005 for an example of surface configurational entropy treatment). In this case, the relative free energy Δ*G*_slab_ in Equation (44) is replaced by the total energy differences. In order to construct the phase diagram, a range of *N*_O_ is considered, and for each value of *N*_O_ several surface structures are calculated. The *N*_O_ and the surface structure that minimize Δγ (Equation 44) yield the equilibrium composition and surface structure at given (*T, p*), provided the surface structure with the lowest energy is among the calculated ones. The resulting phase diagram is shown in Figure 8.

Since we neglected the vibrational free energy and configurational entropy contributions, as well as the *pV* terms, the dependence of Δγ on *T* and *p* enters only through Δμ_O_(*T, p*), and the surface free energy is just a linear function of Δμ_O_(*T, p*), as can be seen from Equation (44). If these contributions are taken into account, additional dependence on (*T, p*) will appear via Δ*G*_slab_. Note that the sharp transitions from one surface state to another (e.g., the crossing between the lines in Figure 8, signifying the transition from the clean surface to the surface oxide phase) are an artifact of neglecting the configurational entropy contribution. At a finite temperature, the two or more quasi-degenerate surface configurations with different atomic structures and maybe also *N*_O_ will coexist at the surface, and the transition from one dominant structure to another will be smooth. Qualitatively, the deviation from the straight line in the vicinity of the crossing in Figure 8 will decay exponentially away from the crossing and will be noticeable in a larger range of Δμ_O_ at higher temperatures since the ratio of surface areas occupied by one or another structure is proportional to the Boltzmann factor $\text{exp}\left[\left(\Delta \gamma^{\left(1\right)}-\Delta \gamma^{\left(2\right)}\right)/k_{\text{B}}T\right]$ (the superscripts 1 and 2 denote the two surface structures for which the Δγ(Δμ_O_) lines cross).

**Figure 9:**
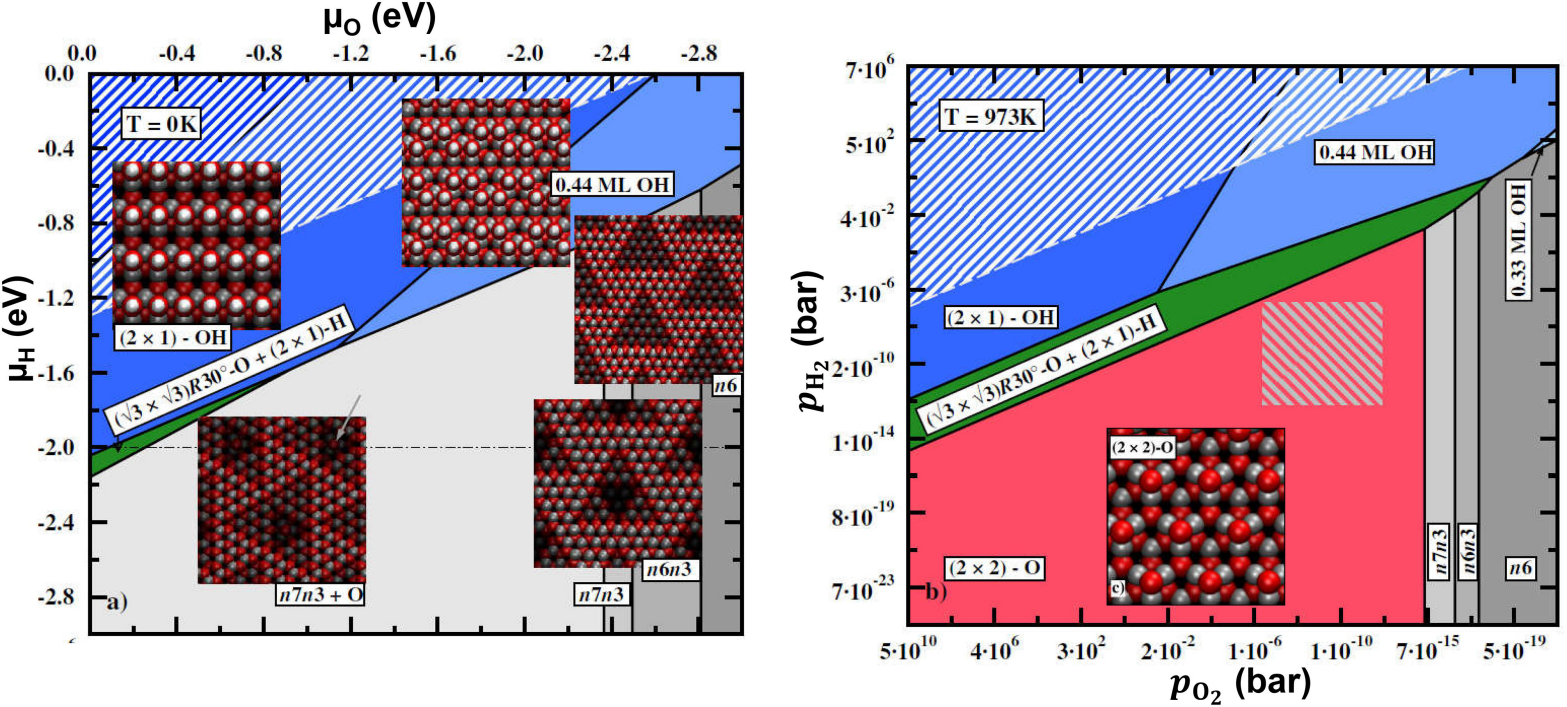
Calculated phase diagrams for ZnO(0001)-Zn surface under an O_2_/H_2_O atmosphere without (left) and with (right) vibrational contributions to the free energy. The vibrational contributions are calculated at *T* = 973 K. Zn, O, and H are denoted by gray, red, and white spheres, respectively. Reproduced from Valtiner et al. (2009) with permission from APS.

Similar considerations apply to more complex situations when the atmosphere consists of more than one particle type. In this case, the surface free energy will depend on chemical potentials of several species:

(45) $$\begin{array}{l} \Delta \gamma \left(T,\left\{p_{i}\right\},\left\{N_{i}\right\}\right)=\frac{1}{A}\left(\Delta G_{\text{slab}}\left(T,p,\left\{N_{i}\right\}\right)-\underset{i}{\sum }N_{i}\Delta \mu_{i}\left(T,p_{i}\right)\right), \end{array}$$

with $p=\underset{i}{\sum }p_{i}$. The surface energy Δγ becomes a hyperplane in the space of Δμ_*i*_. In the case of two gas-phase species, different surface compositions/structures are represented by different (possibly crossing) planes. A 2D surface phase diagram is obtained by projecting the parts of the planes corresponding to the lowest surface free energy at given (μ_1_, μ_2_) onto a Δγ(μ_1_, μ_2_) = const plane. This will create a map with regions in the (μ_1_, μ_2_) space signifying different surface phases. An example of the 2D phase diagram is shown in Figure 9. As in the case of a single gas-phase species, the sharp borders between the phases resulting from the projection of the plane crossings are in reality smoothed out by configurational entropy.

Although thermodynamic equilibrium in general implies equilibrium between all parts of the system, there are technologically important situations when the gas-phase equilibrium is too slow to be reached in a reasonable time, while the equilibrium between all gas-phase components and the surface is reached relatively quickly. In such cases, a constrained equilibrium approach (Reuter and Scheffler, 2003a,b) can be employed.

While in some cases neglecting vibrational contributions to the free energy can be justified, this is not true in general. As was demonstrated, e.g., in Valtiner et al. (2009), taking into account vibrational contributions can result not only in quantitative but also in qualitative changes in the phase diagram, when the relative stability of different phases is reversed by the vibrational contributions. Figure 9 left panel shows the phase diagram of the polar ZnO(0001)-Zn surface under an O_2_/H_2_O atmosphere when the vibrational contributions are neglected, while in Figure 9 right panel they are included. The most striking effect of including vibrational contributions is the appearance of the new phase (the 2×2-O phase) on the diagram. This makes the calculated phase diagram consistent with the experimental measurements (Valtiner et al., 2009).

## 6. Conclusions

In this tutorial review, we have briefly discussed existing and emerging methods for evaluating thermodynamic properties of solids from first principles. As practical examples, approaches to modeling thermodynamics of alloys, crystal surfaces, and point defects at realistic temperature and pressure conditions are discussed in detail. In particular, we introduced methods for calculating configurational entropy and related thermodynamic properties. To evaluate configurational entropy, configurational DOS must be calculated. Approaches for the efficient calculation of configurational DOS are thus also discussed in detail. In particular, recently developed Wang-Landau and nested sampling algorithms are introduced. These approaches allow for calculating configurational DOS without the need for sampling configurations at each temperature.

A fast but accurate evaluation of relative energies of numerous atomic configurations for a given system is required for calculating the configurational contributions to the free energy. We show how cluster expansion based on DFT data can be used to achieve the necessary speed and accuracy. This approach is, however, only applicable to systems whose configurations can be represented by site occupations of a lattice. More general situations (for example, when configurational changes include strong atomic relaxations and reconstructions) require development of more flexible methods for interpolating potential-energy surfaces. Particularly promising in this regard is the development of machine-learned interatomic potentials learning (Behler and Parrinello, 2007; Rupp et al., 2012; Bartók et al., 2013; Li et al., 2015; Shapeev, 2016; Huo and Rupp, 2017), which are unbiased in terms of functional form and can be systematically improved by increasing the number of training data. These approaches are relatively new, however, and have been so far applied to a limited number of system types. Further development and testing of these methods are necessary to insure reliability across chemical space.

Despite the ubiquitous nature and important consequences of statistical effects, in particular configurational sampling, the account for these effects in a rigorous and system-dependent way in modeling remains rare. This is partly because their role is still often underestimated. We hope that this review will help computational materials scientists to appreciate more the role of statistical effects at realistic temperatures, and of configurational entropy in particular, and to quickly start with including these effects into their models.

## Author Contributions

CS and SL contributed equally to the design and writing of this article.

## Conflict of Interest

The authors declare that the research was conducted in the absence of any commercial or financial relationships that could be construed as a potential conflict of interest.

## Footnotes

^1^There is, however, a normalization factor *N*! due to the invariance of the translational states of the whole system to the permutation of the identical molecules
